# The Effects of Formulation on Imidacloprid Dissipation in Grapes and Vine Leaves and on Required Pre-Harvest Intervals under Lebanese Climatic Conditions

**DOI:** 10.3390/molecules27010252

**Published:** 2021-12-31

**Authors:** Liliane Majed, Salem Hayar, Rawan Zeitoun, Britt Marianna Maestroni, Sylvie Dousset

**Affiliations:** 1 Doctoral School of Science and Technology, Platform for Research and Analysis in Environmental Science (EDST-PRASE), Rafic Hariri Campus, Hadath-Baabda 1003, Lebanon; liliane.majed@univ-lorraine.fr (L.M.); rawan.zeitoun@gmail.com (R.Z.); 2 Laboratoire Interdisciplinaire des Environnements Continentaux, Université de Lorraine–CNRS, Bd des Aiguillettes, BP 70239, 54506 Vandœuvre-lès-Nancy, France; sylvie.dousset@univ-lorraine.fr; 3 Department of Plant Protection, Faculty of Agricultural Engineering and Veterinary Medicine, Lebanese University, Dekwaneh-Matn 90775, Lebanon; 4 Environmental Health Research Lab (EHRL), Faculty of Sciences, Section V, Lebanese University, Nabatieh 1700, Lebanon; 5 Department of Chemistry and Biochemistry, Faculty of Sciences, Section V, Lebanese University, Nabatieh 1700, Lebanon; 6 Food and Environmental Protection Laboratory, Joint FAO/IAEA Centre of Nuclear Applications in Food and Agriculture, Department of Nuclear Sciences and Applications, International Atomic Energy Agency, Wagramerstrasse 5, A-1400 Vienna, Austria; B.M.Maestroni@iaea.org

**Keywords:** imidacloprid, vine leaves, grape, QuEChERS, SL and WDG formulation, dissipation, half-life, pre-harvest intervals

## Abstract

In this study, imidacloprid, a systemic insecticide, currently having a specified European Commission MRL value for vine leaves (2 mg kg^−1^), was applied on a Lebanese vineyard under different commercial formulations: as a soluble liquid (SL) and water dispersible granules (WDG). In Lebanon, many commercial formulations of imidacloprid are subject to the same critical good agricultural practice (cGAP). It was, therefore, important to verify the variability in dissipation patterns according to matrix nature and formulation type. Random samplings of grapes and vine leaves were performed starting at 2 days until 18 days after treatment. Residue extractions were performed according to the QuEChERS method and the analytical determination using liquid chromatography coupled to tandem mass spectrometry (LC-MS-MS). The SL formulation yielded significantly higher initial deposit than the WDG formulation on grapes and vine leaves. The formulation type did not significantly affect the dissipation rates; the estimated half-lives in grapes and vine leaves were 0.5 days for all imidacloprid formulations. No pre-harvest intervals were necessary on grapes. PHIs of 3.7 days for the SL formulation and 2.8 days for the WDG formulation were estimated on vine leaves. The results showed that the type of formulation and the morphological and physiological characteristics of the matrix had an effect on the initial deposits, and thus residue levels, but not on the dissipation patterns.

## 1. Introduction

Grapevines are cultivated all over the world, yielding a wide range of products that are part of our daily diet. Grapes, the most economically important product, can be used to make juice, jellies, wine, and pies, and the leaves can be used in cooking [1,2,3]. In Lebanon and nearby countries, vine leaves are commonly used in the preparation of several traditional dishes, especially the famous dishes in “Lebanese Mezze”. As with any other plant, grapevines are vulnerable to fungal and pest infestation and thus the use of phytosanitary products may be unavoidable in order to prevent and control any occurring disease to increase the yield [1,3].

However, the use of these products may be harmful to final consumers, since they could be exposed to residues of phytosanitary molecules through their daily diet [4]. To overcome this challenge and make good use of pesticides without compromising human and environmental health, national and international bodies, mainly the European Commission (EC) and Codex Alimentarius, have specified legal limits for residues in food, i.e., maximum residues limits (MRLs).

Ensuring that residues are below MRLs is of high importance for producers to meet regulatory and market requirements. Yet such a goal may be impossible to attain for some crops due to lack of specific MRLs. This the case for vine leaves, for which no specified legal limits for pesticide residue levels have been set by national and international organizations; as a consequence, an MRL that corresponds to the limit of detection (LOD) of the analytical method for the molecules applied on grapevines is assigned for this commodity, i.e., at the European Union level [5,6,7,8,9].

In the field, MRLs are the benchmark against which it is possible to set the preharvest interval values (PHI). PHI corresponds to the time gap between pesticide applications and the crop harvest in order to yield a healthy product that is in compliance with the legal limit [3]. Many studies concluded that the molecules’ physiochemical properties, the formulation properties, the local climatic conditions, and the plant physiology could affect the main two parameters used in pesticide residues studies, i.e., dissipation rates and PHIs [3,10,11,12]. That is why any possible factor affecting these two previously mentioned parameters must be investigated in order to identify the different variables involved and to gain a better understanding of their interactions.

Formulating a pesticide is about combining an active ingredient with compatible “inerts” or “inactive ingredients”. Inerts are present to achieve specific results; they can be emulsifiers, petroleum solvents, wetting agents or UV-light blocking chemicals, etc., that increase the persistence of active ingredients and enhance their application and performance [13]. Throughout the industry, pesticide products are marketed as emulsifiable concentrates (EC), microencapsulated formulations (ME), flowable (F), water dispersible granules (WDG), sprayable (S), wettable powders (WP), among others. The type and amount of inert ingredients give the phytosanitary product its uniqueness and thus allows distinction between phytosanitary product lines and markets. Therefore, when selecting which formulation to use, farmers must take into consideration the potential influence of formulants on pesticide efficacy, and more importantly, their potential impact on residues level in crops [14], which is one of the biggest concerns for producers. In fact, non-conclusive results can be found throughout the literature pertaining to the effect of formulation type on residues dissipation. Cabras et al. [15] stated that liquid formulations yield more residues compared to granulated ones and Abdel-Hamid et al. [11] correlated the initial deposit of pesticides on tomato fruits to the variation of physical and chemical properties among pesticide formulations. They demonstrated that EC formulations of fenpyroximate showed higher persistence compared to suspension concentrate (SC) formulations on tomato fruits, likewise for imidacloprid, where they compared four different formulations (SC, WDG, SL and WP) and found lower initial deposits with higher degradation rates for the SC formulation compared to the others [11]. Buzzetti [16] also demonstrated that the pesticide formulation of acetamiprid, imidacloprid and diazinon had an effect on the initial pesticide deposit and persistence on apple samples, but not in the case of l-cyhalothrin. Montemurro et al. [17] compared three different formulations of chlorpyrifos and showed different dissipation rates for EC and WG formulations as compared to ME formulation in orange fruits; however, surprisingly, they observed a similar behavior for the three formulations (EC, WG and ME) in orange leaves and soil. In contrast, after conducting four comparative dissipation studies of three commercial formulations of penconazole 10% EC on four varieties of tomatoes, Abou Zeid et al. [18] concluded that there was no statistically significant difference in rate of dissipation among the three evaluated EC formulations. Similarly, Alister et al. [12] concluded that formulation type (SC, SL and WP) did not have a significant effect on initial deposit and dissipation rates of acetamiprid, buprofazine and fenhexamid on apple fruits and grape berries.

Imidacloprid, 1-(6-chloro-3-pyridylmethyl)-*N*-nitroimidazolidin-2-ylideneamine, a predominantly systemic insecticide, is extensively used for the control of a wide range of insects and pests at various stages of grape cultivation especially thrips and mealybug [19]. It is important to note that imidacloprid is no longer approved for use by the European Commission since 1 December 2020, according to EU resolution EU/2020/1643 [20]. Starting June 2022 import tolerances will be applied, and the applicable MRLs will be 0.7 mg kg^−1^ and 0.01 mg kg^−1^ for grapes and vine leaves respectively; the latter value corresponds to the lower limit of analytical determination for vine leaves [20]. These new MRLs will be replacing the currently approved MRLs of 1 mg kg^−1^ and 2 mg kg^−1^ for grapes and vine leaves, respectively (Reg. (EU) No 491/2014) [21]. As stated by the EU pesticide data base, these modifications were not implemented due to toxicological concerns, but rather due to unavailability of data. In Lebanon, 16 commercial formulation products, registered under different trade names, contain imidacloprid as the main active ingredient. They are subject to the same critical good agricultural practice (cGAP), that is, the same PHIs and the same application rates, despite the fact they differ in the composition of co-formulants [18]. It was, therefore, important to study the variability of the imidacloprid formulation type as soluble liquid concentrate (SL) or as water dispersible granules (WDG) on the dissipation rates and the PHIs on grapes and vine leaves under Lebanese climatic conditions.

## 2. Results and Discussion

### 2.1. SL-and WDG-Imidacloprid Dissipation Kinetics

**Figure 1:**
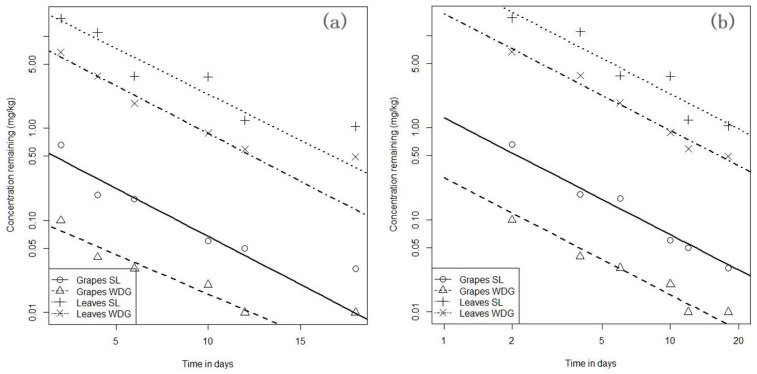
(**a**) Plot of residual Imidacloprid on a linear-log scale using a first order decay model. (**b**) Continuous change model of imidacloprid degradation with pooled slope but individual intercepts.

The statistical analysis of imidacloprid residues concentration data showed that the first order decay model according to Equation (1) was a useful approximation of the data up to day 12 (Figure 1a). The data relative to day 18 data did not fit the first order decay model. Therefore, a two-compartment model was formulated; however, there were insufficient data to establish the point of change from the first to the second compartment as well as the rate of decline in the second compartment. Therefore, an alternative statistical model, called the continuous change model, was proposed to fit the data. In this model, the half-life is steadily increases with time. The rate of imidacloprid dissipation after day 12 is very slow, but still occurring according to the model. Such a model was considered satisfactory (Figure 1b), see also Figures S1 and S2 and Tables S1 and S2 in the Supplementary Materials. In this model the slopes of the fitted lines did not differ significantly from each other. The pooled slope of the regression lines (corresponding to the Kdiss) was −1.269 ± 0.068. Significant differences were found for the regression intercepts as shown in Table 1.

### 2.2. Matrix and Residue Levels

Regardless the formulation type, imidacloprid residues were found to be 20 to 70 times higher in vine leaves than in grapes at all sampling times for all of the analyzed samples (Table 2). The literature relates these finding to the morphological and physiological differences between vine leaves and grapes and to the fact that grapes are covered by the leaves i.e., greater contact surface.

According to Edwards [22], the distribution, retention and ab/adsorption of pesticides in/on plant tissues are greatly influenced by plant morphological and physiological characteristics. In addition, Maclachlan and Hamilton [10] stated that complex factors dictate the quantity of pesticide initially deposited and retained on leaves, i.e., their nature, the phytosanitary molecules’ proprieties and abiotic factors such as wind speed, temperature and humidity. Maclachlan and Hamilton [10] also underlined the importance to take canopy density and crop leaf surface into consideration when it comes to spray deposits, given that the canopy acts as a filter of spray droplets and thus deeper parts of the plant far from spray nozzle may receive less spray. 

Furthermore, Lichiheb et al. [23] and Fernández and Eichert [24] mentioned that leaf cuticle (permeability of leaf surface) and pesticide lipophilicity are two of the main factors influencing pesticide penetration in plants. Possingham et al. [25] studied wax structure and composition of leaves and fruit of Vitis vinifera and found a “considerable qualitative difference between the waxes of leaves and fruits”, where grapes’ cuticular wax consisted of a “hard” wax component (70%); i.e., oleanolic acid; and a “soft” wax component, i.e., mixture of long chain acid, alcohols, aldehydes, ester and hydrocarbons; meanwhile, leaves had only the “soft” fraction.

Since diffusion is the main process for insecticide penetration [26], cuticular waxes affect that process by reducing solutes mobility [27] and pesticide transfer is driven by its lipophilicity and concentration [3]. It is thus harder for molecules with low Kow (logP = 0.57) and high water solubility (610 mg kg^−1^), such as imidacloprid [28], to move through grapes’ than through leaves’ cuticular waxes, which explains higher residues found in leaves compared to grapes regardless of leaves’ density and vines’ conducting system (pergola).

Hence, our results underline the impact of plants’ nature and morphology on the amount and distribution of residues across plant parts and are in agreement with results obtained by Alister et al. [12], Bletsou et al. [29], Abdallah [30] and Hanafi et al. [31]. Bletsou et al. [29] showed the effect of leaf density, where they used higher application rates of bifenthrin in beans (2.9 kg ha^−1^) than in peas (2.2 kg ha^−1^), and found 2.5 times less initial deposit on green beans compared to peas. This result was related to morphological structure differences as green beans did not receive most of the spraying solution due to coverage by their leaves, while pea pods, having smaller leaves, were almost totally exposed to spraying [29].

As in our paper, Abdallah [30] also found higher residues of chlorfenapyr and difenoconazole in vine leaves compared to grapes. Cuticular wax chemistry and structural arrangement, which influence pesticide penetration [26,32], change according to fruit type and growth stage. Alister et al. [12] endorsed the effect of cuticular wax on pesticide penetration, where they concluded that fruit growth stage was the predominant parameter affecting pesticide initial deposit and dissipation rate, and that the effects of environmental parameters, such as rain, are important to consider; however, ultimately, it is the fruit type that determines the amount of pesticide penetration. Finally, Hanafi et al. [31] used the same application rate of imidacloprid (0.625 kg ha^−1^) and oxamyl (1.8 kg ha^−1^) on green beans and chili peppers and found residue level for both molecules higher in green beans compared to chili peppers; similarly, they attributed these findings to morphological characteristic of each plant and to the so-called “dilution-effect” related to the growth stage.

### 2.3. Formulation and Residues Level

As shown in Table 2, despite a lower application amount per unit area (0.07 kg ha^−1^ for SL and 0.21 kg ha^−1^ for WDG), higher initial residues levels were found in vine leaves and grapes treated with SL- imidacloprid compared to WDG- imidacloprid. The finding that the SL formulation yielded more residues than the WDG formulation is in accordance with the results obtained by Buzzetti [16], where, in her work on apples, higher residue levels, initial and final deposits, of imidacloprid were found when applied as SL formulation (initial: 1.20 mg kg^−1^, final: 0.47 mg kg^−1^) compared to WP (initial: 0.90 mg kg^−1^, final: 0.30 mg kg^−1^) and soluble concentrate (SC) (initial: 0.89 mg kg^−1^, final: 0.29 mg kg^−1^) formulations. The author inferred that the variations of the ratio and nature of the other components of the formulated product (adjuvants, surfactant, inerts…) were behind the variation of the level of residues detected between SL, WP and SC formulations, despite the fact that all the treatments were performed in a way to obtain the same dose of active ingredient per hectare [16]. Moreover, Buzzetti [16] explained the similarity of the level of residues of the WP and SC formulations to be due to the fact that both have in common that they form suspensions on water compared to the SL formulation that forms a solution. This is a similar situation as the study described in this paper where a SL formulation and another suspension forming formulation (WDG) are compared.

Abdel-Hamid et al. [11] also reported a great influence of the formulation type on the residue level, more precisely on the initial deposits, when comparing 4 different formulations (SL, WDG, SC and WP) of imidacloprid in their two consecutive year study (2009 and 2010). However, and contrary to Buzzetti [16] and with the results presented in this study, among the four formulations they studied, they reported higher residues level in tomatoes for imidacloprid WDG formulation (initial: 4.55 mg kg^−1^ in 2009 and 3.68 mg kg^−1^ in 2010, final: 0.51 mg kg^−1^ in 2009 and 0.30 mg kg^−1^ in 2010) compared to the SL formulation (initial: 3.11 mg kg^−1^ in 2009 and 2.49 mg kg^−1^ in 2010, final: 0.05 mg kg^−1^ in 2009 and bellow the detection limit in 2010) [11]. Taken together, these findings support the hypothesis that the formulation type has an impact on the level of residues on vine leaves and grapes according to the Food and Agriculture Organization (FAO) statement: “While having the same concentration of an active ingredient two products are not considered similar if they have different formulations or have different synthetizing methods” [16].

The dissipation rates (k) of SL and WDG formulations are shown in Table 1. Despite the previously discussed higher initial deposits of the SL formulation compared to WDG’s, and the higher residues found on vine leaves than on grapes, the two formulations followed the same dissipation patterns and had quite similar dissipation rates of 1.269 day^−1^ on grapes and vine leaves, leading to similar half-lives of 0.5 day for the two formulations.

Pre-harvest intervals were estimated according to the MRLs set by the European Commission on vine leaves and grapes (Table 1). For grapes, no PHIs were necessary since all estimated PHIs values were less than one day for the two formulations, which could be related to the aforementioned low initial deposits of imidacloprid on grapes. In the case of vine leaves, when using the currently applicable EU MRL (2 mg kg^−1^) for the calculations, the calculated PHIs were 2.4 and 1.7 days for the SL and the WDG imidacloprid formulation, respectively. Whereas, when the new EU MRL (0.01 mg kg^−1^) that is approved for application starting June 2022 was used, the PHIs were 6.6 and 5.9 days for the SL and the WDG imidacloprid formulation, respectively.

Furthermore, it was noticeable that after only 12 days post-treatment nearly 90% of imidacloprid residues had dissipated in grapes and vine leaves for the two formulations (Table 2), which is consistent with previous studies where 98% of imidacloprid dissipated after 6 days in sugar beet and where total imidacloprid dissipation was observed after 15 days in broad bean [11]. Likewise, imidacloprid rapid dissipation was widely discussed in the literature and short PHIs were reported in various matrix, e.g., vine leaves and grapes, tomatoes, okra, rocket, parsley, green beans, chili peppers, zucchini, etc. [11,19,30,31,33,34,35]. It was found to be due to imidacloprid’s high sensibility to photodegradation, even under low light intensity conditions [36,37]. Altogether, these studies demonstrated a significant PHI dependence on climatic conditions (sunlight, humidity, temperature, etc.) and they underlined the need to determine PHIs on a regional scale to ensure their accuracy and reliability.

## 3. Materials and Methods

### 3.1. Chemicals and Reagents

Analytical imidacloprid standard was purchased from Dr. Ehrenstorfer. Analytical grade solvents and reagents, acetonitrile, methanol and ammonium acetate were purchased from Sigma-Aldrich International GmbH (Munich, Schnelldorf, Germany). Laboratory ultra-pure water was obtained using Milli-Q water purification system (Millipore, Billerica, MA, USA). NaCl, anhydrous MgSO4, PSA, and GCB were purchased from Agilent technologies (Santa Clara, CA, USA). The two commercially formulated imidacloprid products used in field trials Diclean 20% (SL) and Pilarking Plus 70% (WDG) were officially registered in the Ministry of Agriculture of Lebanon and were purchased form Amalia, S.A.L., Verdun, Rabah Center 5th floor, Beirut, Lebanon and the National Development and General Trading Co., Bank Street, Tyre, Lebanon (Table 3).

### 3.2. Site Location and Specification

The vineyard of local Tfeifihi variety (Vitis vinifera) (1200 vines–12 years old–conduction system: pergola) was located in Tamnine-El-Tahta, Governorate of Baalbeck Hermel [33°52′43.8″ N 36°00′13.9″ E] at an altitude of 960 m and has an area of 5000 m^2^. No imidacloprid treatments were performed on the target vineyard before the study.

### 3.3. Pesticide Application and Sampling

Imidacloprid was applied in the first week of July 2018, when the temperature was 31 °C with passing clouds and the wind speed was 2 km/h blowing from 270° West to East with a relative humidity of 39%. During the sampling period, the temperature varied between 18 and 32 °C, the relative humidity ranged between 27 and 65% and no precipitations were recorded.

**Figure 2:**
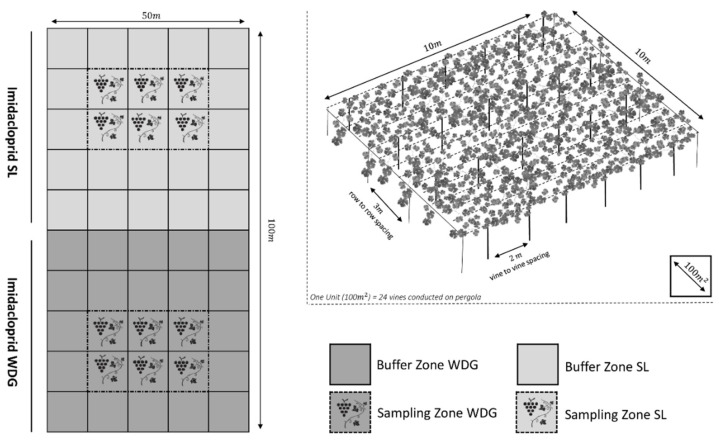
On the left: layout of the field experimental design showing the surface area treated with imidacloprid SL (light grey), the surface area treated with imidacloprid WDG (dark grey), buffer zones and sampling zones. On the top right: an overview of vines conduction system (pergola) and canopy density per unit.

As shown in Figure 2, the field was divided into two equal plots of 2500 m^2^, each plot was treated according to the OECD guidelines for crop field trials [38], with one imidacloprid commercial product per plot, Diclean 20% (SL, rate 0.35 L ha^−1^) and Pilarcking Plus 70% (WDG, rate 0.30 L ha^−1^). A back sprayer calibrated according to FAO guidelines on good practice for ground application of pesticides (nozzle calibrated to 200–400 µm with a spray pressure of 40 PSI) was used for applications [39]. A buffer zone of 2000 m^2^ consisting of treated but unsampled vines was established to separate the sampling zones (1200 m^2^ ~ 144 vines) delimited in each plot. Label indications were followed meticulously to prepare the imidacloprid formulations and extra care was taken to make sure the products were homogeneously dissolved. For WDG treatments, continuous agitation of the tank was done during spraying to keep the imidacloprid ingredient suspended in water.

#### Sampling Procedure for Grapes and Vine Leaves

Randomized sampling of vine leaves was conducted every 2 days, from 2 days up to 18 days after treatment. Sampling was implemented according to FAO guidelines (CAC/GL 33–1999) [40]. For each sampling date, one composite field sample of 2 kg of vine leaves and grapes was collected. From the composite sample a laboratory sample of 1 kg was subsampled and weighed, kept in polyethylene bag and sent directly to the laboratory for residue analysis. The laboratory sample (1 kg sample) was homogenized using a VCM4 Waring Vertical Cutter Blender/Mixer 309 (Hallde, Sweden) and 5 replicate analytical portions of 10 g were taken for analysis.

### 3.4. Residue Extraction and Clean-Up

Residue extraction was performed following the original unbuffered QuEChERS method, which is widely used for pesticide residue extraction. QuEChERS is an abbreviation for quick, easy, cheap, effective, rugged and safe, and it was developed and first published by Anastassiades et al. [41]. Ten grams of a homogenized sample were weighed in a 50 mL polypropylene centrifuge tube, 10 mL of acetonitrile (ACN) were added. The mixture was shaken by hand for 1 min, followed by addition of 4 g of MgSO_4_ and 1 g of NaCl. The tube was manually shaken again for 1 min. Afterwards, the tube was subjected to centrifugation, for 10 min at 2066 g. One ml of the supernatant was isolated and put in a dispersive solid-phase extraction (d-SPE) tube containing 150 mg MgSO4, 25 mg primary secondary amine (PSA) and, only for vine leaves samples, 50 mg graphitized carbon black (GCB). The tube was shaken for 1 min and then subjected to centrifugation for 10 min at 3000 rpm. The extract was isolated in a 15 mL polypropylene tube and put in the refrigerator overnight. Finally, the supernatant was filtered using a 0.20 μm PTFE filter. Different levels of dilutions (100 times and 200 times dilution) were performed in acetonitrile in order to minimize the matrix effect and to reduce the concentration level to a level that would fall within the validated analytical range. The final extract was transferred into a glass vial to be directly analyzed by liquid chromatography mass spectrometry (LC-MS/MS).

### 3.5. Instrumentation and LC-MS/MS Analytical Conditions

**Figure 3:**
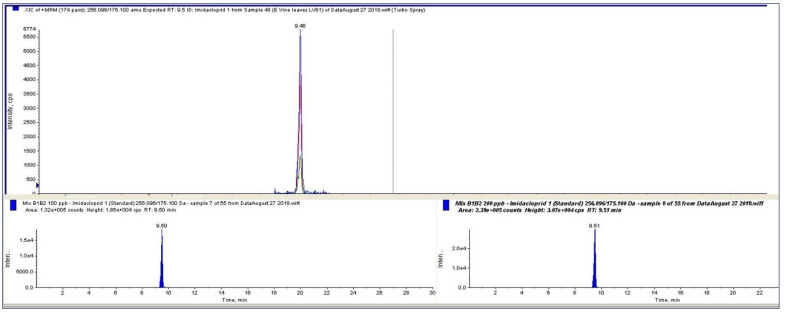
Total ion chromatogram (TIC) of the MRM of imidacloprid and the extracted ion chromatogram for imidacloprid in vine leaves at 100 μg/L (lower left figure) and 200 μg/L (lower right figure).

The LC-MS/MS analysis was performed using Agilent Technologies 1200 Infinity Series liquid chromatograph coupled to 3200 QTrap Triple Quadrupole Mass Spectrometer (AB Sciex, Dublin, CA, USA). The unit was equipped with a Phenomenex Analytical, C18 Synergi Fusion 150 × 0.25 mm × 2.5 μm, separation column and a guard column. The injection volume of 5 μL was delivered using an automatic injector with a flow rate of 0.4 mL/min. The eluent was composed of a solvent water (A)-methanol (B) gradient (MeOH), which was buffered with 5 mM ammonium acetate. The gradient program was as follows: 2% B to 100% of B over 12 min, held at 100% B until 20 min then decreased to 2% B at 25.01 min. The total run time was 30 min. The retention time of imidacloprid was 9.47 min. The equipped mass spectrometer provides the capability of combining positive and negative ionization modes by ESI. It was operated in positive ion mode; MRM (multiple reaction monitoring) mode was used for data acquisition (Figure 3). 

Table 4 shows the optimized parameters used for imidacloprid qualification and quantification. The source temperature and the ion spray voltages were 500 °C and 5000 v respectively. The ions underwent fragmentation by collisions with nitrogen (inert gas) that was also used as nebulizer curtain gas. Pre-configured iMethod™ Application (AB Sciex) and associated libraries designed for quantitative and qualitative screening using QTRAP^®^ technology were used. EU SANTE/12682/2019 guidelines were followed for imidacloprid identification and quantification [42].

### 3.6. Method Validation for Grapes and Vine Leaves

Method validation was implemented according to Hayar et al. [7]. The following parameters, as required by EU SANTE/12682/2019 [42], were established: linearity (R^2^), recovery (RM%), within-laboratory repeatability (RSD_r_%) and reproducibility (RSD_RW_%) and the limit of detection and quantification (LOD and LOQ, respectively).

As per Hayar et al. [7], linearity was performed by first preparing a stock solution of 1000 mg kg^−1^ of imidacloprid standard in acetonitrile. Afterwards, aliquot solutions were obtained by serial dilution with 6 concentrations ranging from 5 to 500 µg kg^−1^. These solutions were later used to build standard calibration curves. Similarly, matrix-matched standard solutions were prepared by adding an imidacloprid standard to blank sample extracts, previously prepared, of grapes and vine leaves. Linear regression of all calibration curves had regression coefficient R2 greater than 0.99. The limits of detection and quantification (LOD and LOQ) were 1.93 and 6.45 µg kg^−1^, respectively, in vine leave matrix. In grape matrix, the limits of detection and quantification (LOD and LOQ) were 1.08 and 5.03 µg kg^−1^, respectively.

Recovery and % RSD were determined by fortifying matrix blanks (10 g) with three concentration levels (0.01; 0.05; and 0.1 mg kg^−1^) of imidacloprid standard mixture. Five replicates of each fortification level were prepared on three different days. After fortification, the samples were left at room temperature for 30 min to allow the pesticide to be evenly incorporated into the matrix. Later, QuEChERS extraction procedure was performed and followed by LC-MS-MS analysis, as described in Section 2.3, respectively.

For the method to be satisfactory for imidacloprid analysis, EU SANTE/12682/2019 guidelines [42] require recovery values between 70% and 120% with a relative standard deviation (% RSD) less than 20%, for samples tested on the same day (expressed as repeatability % RSD_r_) and for samples analyzed on three different days (expressed as reproducibility % RSD_RW_). In our study, recovery means were greater than 92% and 80% for grapes and vine leaves, respectively, with RSD % < 20% for all values (Table 5).

### 3.7. Statistical Analysis

The data was subjected to statistical analysis using R free software [43] to give regression equations and half-life *(DT_50_)* (Table 1 and Table 2) and was fit to a first order kinetic dissipation model (Maclachlan and Hamilton [10]) according to Equation (1):

(1) $$C_{t}=C_{0}e^{-kt}$$

where *C_t_* represents the residual concentration at sampling time t, *C_0_* represents the initial concentration and *k* represents the dissipation rate of the molecule and at the same time the slope of the exponential regression curve that is used for the determination of the half-life which is the time required for imidacloprid to decrease to half of its initial concentration after application [3]. The following Equation was used:

(2) $$DT_{50}=\frac{ln2}{k}$$

Pre-harvest intervals *(PHI)* were estimated as the time needed for the residues to fall to their specified EU MRL (see Table 1) and were derived from Equation (3) PHIs were estimated as the time needed for the residues to dissipate to values equivalent to MRL after pesticide application (time 0) using an established regression model. The Equation used was:

(3) $$PHI=\left[\frac{intercept-ln\left(MRL\;value\right)}{k}\right]$$

For data visualization, R software version 3.6.3 packages were used [43].

## 4. Conclusions

The effect of formulation type on imidacloprid residues in vine leaves and grapes was investigated. Higher residue levels were detected when grapevines were treated with Diclean 20% (SL) than when treated with Pilarcking Plus 70% (WDG). The type of formulation and the morphological and physiological characteristics of the matrix were found to have an impact on initial deposits, and thus on residue levels, but not on the dissipation patterns.

Since each product formulation is unique, the designers of pesticide formulations have a wide territory to innovate out of the traditional basic roles of adjuvants as carriers, penetrants, stickers, buffers, etc., and move towards more holistic approaches when developing new products that encompass all the legal, economical, ecological and safety challenges from farm to fork. Consequently, the improvement in formulation and inert compositions will enable new phytosanitary products to meet regulatory authorities’ requirements, which are becoming more and more restrictive especially when it comes to pesticide residues in food products and safety to applicators. 

Further field studies need to be conducted under Lebanese pedoclimatic conditions in order to set more accurate and reliable PHIs, specific to the local environmental conditions, and to provide farmers with the knowledge they need to choose the appropriate pesticide formulation for their crop variety (e.g., vines, apple), targeted matrix (e.g., berries or leaves), the plants’ growth stage (e.g., grapes and leaves diameters) and local climatic conditions (e.g., temperature, humidity). 

In this context, this work may be considered as a pilot study for other future ones that will involve other phytosanitary molecules used on grapevines and in which the effect of pesticide application frequencies will be evaluated.

## Figures and Tables

**Table 1:** Regression equations, dissipation rates, half-life and estimated PHI according to European Union 2021 MRLs (EU 2021) and to European Union 2022 MRLs (EU 2022) for grapes and vine leaves.

**Grapes**								
**Imidacloprid Formulation**	**Regression Equation**	**Slope (k)**	**Intercept (b)**	**DT_50_** **(Days)**	**PHI (Days)**		**MRL (mg kg^−1^)**	
					**EU 2021**	**EU 2022**	**EU 2021**	**EU 2022**
SL	$y=1.28e^{-1.269x}$	−1.269 (±0.068)	0.249	0.546	0.196	0.477	1	0.7
WDG	$y=0.29e^{-1.269x}$	−1.269 (±0.068)	−1.249	0.546	−0.984	−0.703	1	0.7
**Vine Leaves**								
**Imidacloprid Formulation**	**Regression Equation**	**Slope** **(k)**	**Intercept (b)**	**DT_50_** **(Days)**	**PHI (Days)**		**MRL (mg kg^−1^)**	
					**EU 2021**	**EU 2022**	**EU 2021**	**EU 2022**
SL	$y=43.55e^{-1.269x}$	−1.269 (±0.068)	3.774	0.546	2.428	6.603	2	0.01 *
WDG	$y=17.37e^{-1.269x}$	−1.269 (±0.068)	2.855	0.546	1.704	5.879	2	0.01 *

* Indicates the lower limit of detection.

**Table 2:** Residues of imidacloprid (SL and WDG) in grapes and vine leaves (*n* = 5).

**Imidacloprid Formulation**	**Grapes**					
	**Mean Concentration (±SD) in mg kg^−1^**					
	**T_2_**	**T_4_**	**T_6_**	**T_10_**	**T_12_**	**T_18_**
SL *	0.66 (±0.031) ^a^ (0) ^b^	0.19 (±0.020) (64.1)	0.17 (±0.012) (67.9)	0.06 (±0.003) (88.7)	0.05 (±0.006) (90.5)	0.03 (±0.008) (94.3)
WDG **	0.10 (±0.004) (0)	0.04 (±0.008) (50)	0.03 (±0.001) (62.5)	0.02 (±0.018) (75)	0.01 (±0.001) (87.5)	0.01 (±0.003) (87.5)
**Imidacloprid Formulation**	**Vine Leaves**					
	**Mean Concentration (±SD) in mg kg^−1^**					
	**T_2_**	**T_4_**	**T_6_**	**T_10_**	**T_12_**	**T_18_**
SL	15.60 (±0.960) (8.77)	11.00 (±0.780) (35.7)	3.69 (±0.510) (78.4)	3.64 (±0.501) (78.7)	1.22 (±0.300) (92.8)	1.05 (±0.230) (93.8)
WDG	6.71 (±0.148) (0)	3.68 (±0.580) (42.5)	1.87 (±0.019) (70.8)	0.89 (±0.090) (86.1)	0.59 (±0.210) (90.8)	0.49 (±0.111) (92.3)

^a^ Mean ± standard deviation of five replications. ^b^ Figures in parentheses indicate cumulative % dissipation through time. * SL: soluble liquid ** WDG: water dispersible granules.

**Table 3:** Pesticides active ingredients and phytosanitary commercial products used for the experimental treatment of vines.

Trade Name	Active Substance (%) Formulation Type	Recommended Dose (L ha^−1^–Kg ha^−1^)	PHI (Days)	Supplier Country	Importer
Pilarking^®^ Plus	Imidacloprid 70% WDG	0.3	14	Zhejiang Hisun Chemical Co., LTD Zhejiang, China	Rmaily Trading Est.
Diclean	Imidacloprid 20% SL	0.35	14	Hailir Pesticides and Chemicals Group Co., LTD, Chengyang, China	National Development and General Trading Co.

**Table 4:** Precursor, transition ions and source parameters for imidacloprid residues analyzed by the LC-MS/MS method.

Condition	Content											
Instrument:	Model AB Sciex 3200 QTRAP LC-MS/MS SYSTEM											
Column:	C_18_ column, Phenomenex Analytical Synergi, 150 × 2 mm, 2.5 μm particle size											
Column Flow:	Gradient elution program at 0.4 mL·min^−1^											
Source temperature:	500 °C–5000 v											
Ion Spray- Potential:	Electron Spray Ionization,											
Mode:	Positive Mode											
Molecule	RT (min)	Precursor ion (*m*/*z*)	Transition Q1 (*m*/*z*)	DP	CE	CXP	Transition Q2 (*m*/*z*)	DP	CE	CXP	LOD	LOQ
				(Volts)				(Volts)			(ng/g)	
Imidacloprid	9.47	256	209	51	21	7	175.0	46	25	7	1.93	6.45

RT, retention time; Q1, first quadrupole; DP, declustering potential; CE, collision energy; CXP, collision cell exit potential; Q2 second quadrupole.

**Table 5:** Method validation results showing the average of recovery data (RM%), repeatability (RSD_r_%) and reproducibility (RSD_Rw_%) for imidacloprid at the three fortification levels, 0.01, 0.05 and 0.1 mg kg^−1^ (*n* = 5 at each level) in grapes and vine leaves samples.

Matrix	Level of Spiking (mg kg^−1^)	Recovery Mean (RM%)	Repeatability (RSD_r_%)	Reproducibility (RSD_RW_%)
Grapes	0.01	96.5	16.6	12.1
Vine leaves		92.0	17.0	19.0
Grapes	0.05	92.6	13.3	9.5
Vine leaves		84.0	7.0	8.0
Grapes	0.1	98.5	1.2	2.3
Vine leaves		82.0	11.0	13.0

## Data Availability

The data presented in this study are openly available in this article.

## Supplementary Materials

The following are available online, Table S1: Summary of two compartment models, Figure S1: Two compartmental model for each treatment combination, Table S2: Summary of model of imidacloprid decomposition with pooled slope but individual intercepts, Figure S2: Model of imidacloprid degradation with separate slopes and intercepts.

## Sample Availability

Samples of the compounds are not available from the authors.
