# Supplementary material for: The Effects of Formulation on Imidacloprid Dissipation in Grapes and Vine Leaves and on Required Pre-Harvest Intervals under Lebanese Climatic Conditions
Source: Molecules. 2021 Dec 31;27(1):252. doi: 10.3390/molecules27010252 (PMC8746927; doi:10.3390/molecules27010252)
Supplement: Supplementary file 1 [file molecules-27-00252-s001.zip › molecules-1517264-supplementary.pdf]

molecules

Supplementary Materials

The effects of formulation on imidacloprid dissipation in grapes and vine leaves and on required pre-harvest intervals under Lebanese climatic conditions

Liliane Majed[1,6], Salem Hayar[1,2,3,*], Rawan Zeitoun[1,3,4], Britt Marianna Maestroni[5], Sylvie Dousset[6]

[1] Doctoral School of Science and Technology, Research Platform for Environmental Sciences (EDST-PRASE), Lebanese University, Rafic Hariri Campus, Hadath-Mount Lebanon, Lebanon; liliane.majed@univ-lorraine.fr
[2] Department of Plant Protection, Faculty of Agricultural Engineering and Veterinary Medicine, Lebanese University, Dekweneh-Beirut, Lebanon; salem.hayar@ul.edu.lb
[3] Environmental Health Research Lab (EHRL), Faculty of Sciences, Section V, Lebanese University, Nabatieh, Lebanon; rawan.zeitoun@gmail.com
[4] Department of Chemistry and Biochemistry, Faculty of Sciences, Section V, Lebanese University, Nabatieh, Lebanon
[5] Food and Environmental Protection Laboratory, Joint FAO/IAEA Centre of Nuclear Applications in Food and Agriculture, Department of Nuclear Sciences and Applications, International Atomic Energy Agency, Wagramerstrasse 5, A-1400 Vienna, Austria; B.M.Maestroni@iaea.org
[6] Laboratoire Interdisciplinaire des Environnements Continentaux, Université de Lorraine - CNRS, Bd des Aiguillettes, BP 70239, 54506 Vandœuvre-lès-Nancy, France; sylvie.dousset@univ-lorraine.fr
[*] Corresponding author: Salem Hayar. Doctoral School of Science and Technology, Research Platform for Environmental Sciences (EDST-PRASE), P.O Box. 5, Campus Rafic Hariri, Beirut, Lebanon. Phone: +961-03416364. E-mail: salem.hayar@ul.edu.lb; hayarsalem@gmail.com

Summary: The statistical analysis of imidacloprid residues concentration data is presented in this document. First order decay was a useful approximation of the data up to Day 12 but the day 18 data did not fit the model. A two compartment model was formulated but there were insufficient data to establish the change from the first to second compartment as well as the rate of decline in the second compartment. There was little difference between the Day 12 and Day 18 data. An approximation resolved the difference between the transition time form one compartment and the next as opposed to the rate of decomposition within the second compartment by assuming no decomposition occurred in the second compartment. An alternative model, where the half-life continually increased was proposed. Such a model is simpler in that it only involved a single slope and four intercepts.

1. Data

A summary of the data is given in Appendix A. The data suggest that the 5 concentrations are analysis replicates of the same incubation.

2. Statistical Methods

The data were plotted on a linear-log scale that is typical for first order decay data. The result was a curved plot. A log-log plot produced a geed approximation to a linear result.

3. Results

3.1. Experimental unit

An analysis which assumed there were incubations form which were taken 5 aliquots for assay. Analysis of variance was performed using incubations as ‘main plots’ and within incubations as ‘subplots. The treatment effects were removed and the day effects removed of covariates based on the logarithm of the day. The within incubation had

a residual mean square of 0.0153 whereas the model using the means of the concentrations had a residual mean square of 0.0542. This confirmed that the modelling should be made on the means of the five assays.

3.2. First order decay model

A plot of residual Imidacloprid against day is shown in Figure 1. Plot of residual Imidacloprid on a linear-log scale is shown in Figure 1. There was a steady decline in concentration until Day 12. After Day 12 there was perhaps some loss, but the amount of loss was not statistically significant. The Day 18 data do not fit a first order decay model. The overall the quadratic term to Day 12 was not statistically significant but the quadratic term was negative for each treatment. There was therefore suggestion of curvature between Day 2 and Day 12.

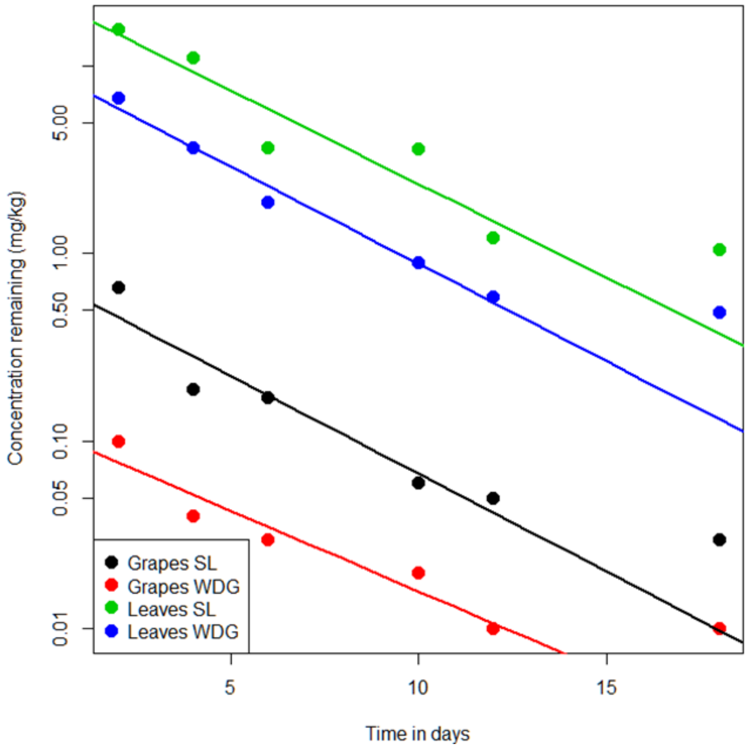

Figure 1. Plot of residual Imidacloprid on a linear-log scale using a first order decay model

3.3. Two compartment model

A plot of two compartment models is shown in Figure S1. The position of the change from one compartment to the other is confounded with the slope or rate of decay in the second compartment. The slope of the second compartment could be defined using information based on the last two observations. Some of those estimates did not have a consistent sign. An approximation, based on the available data, is that there was effectively no decomposition in the second compartment as shown in Figure S1. Details of the model are given in Table S1.

Table S1. Summary of two compartment models

	Intercept	Initial	Half-lives (days)	Last observed (µg/kg)	Duration (days)
Grapes SL	-0.299	-0.240	0.74	2.89	0.03
Grapes WDG	-2.165	-0.198	0.115	3.51	0.01
Leaves SL	3.148	-0.229	23.3	3.02	1.05
Leaves WDG	2.259	-0.238	9.6	2.91	0.49

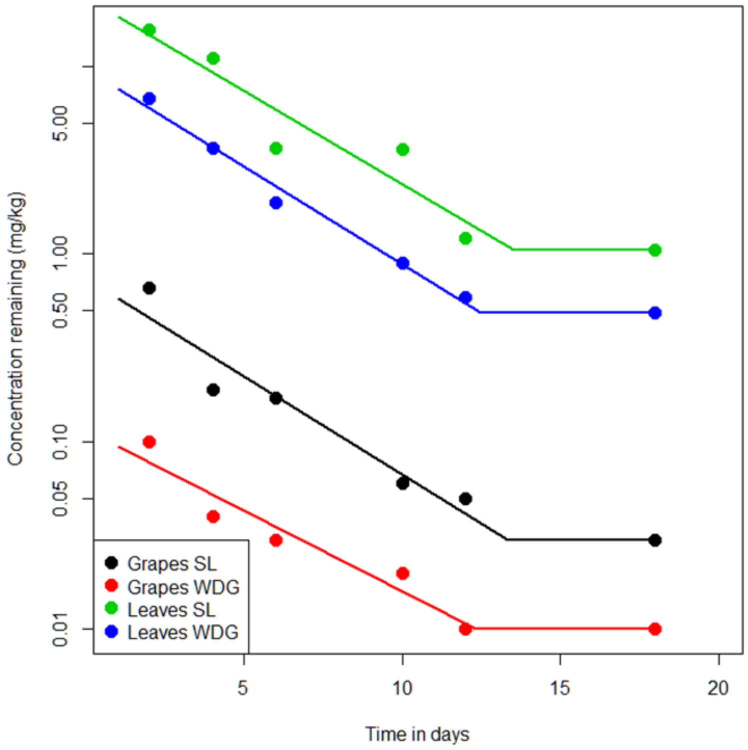

Figure S1. Two compartmental model for each treatment combination

3.4. Continuous change model

A plot on a log-log scale (Figure S2) showed no sign of curvature. This was considered a satisfactory model. There was no significant difference in the slopes between the treatments. This enabled a simplification of the model where there was a common slope and separate intercepts. A summary of that model is given in Table S2.

A plot on a log-log scale (Figure 2) showed no sign of curvature. The slopes of the fitted lines did not differ significantly but there were marked differences in the intercepts. The pooled slope was -1.269 ± 0.068.

Table S2. Summary of model of imidacloprid decomposition with pooled slope but individual intercepts

	**Treatment**	**Estimate**	**Std. Error**
Slope	All	-1.269	0.068
Intercept	Grapes SL	0.249	0.166
Intercept	Grapes WDG	-1.249	0.166
Intercept	Leaves SL	3.774	0.166
Intercept	Leaves WDG	2.855	0.166

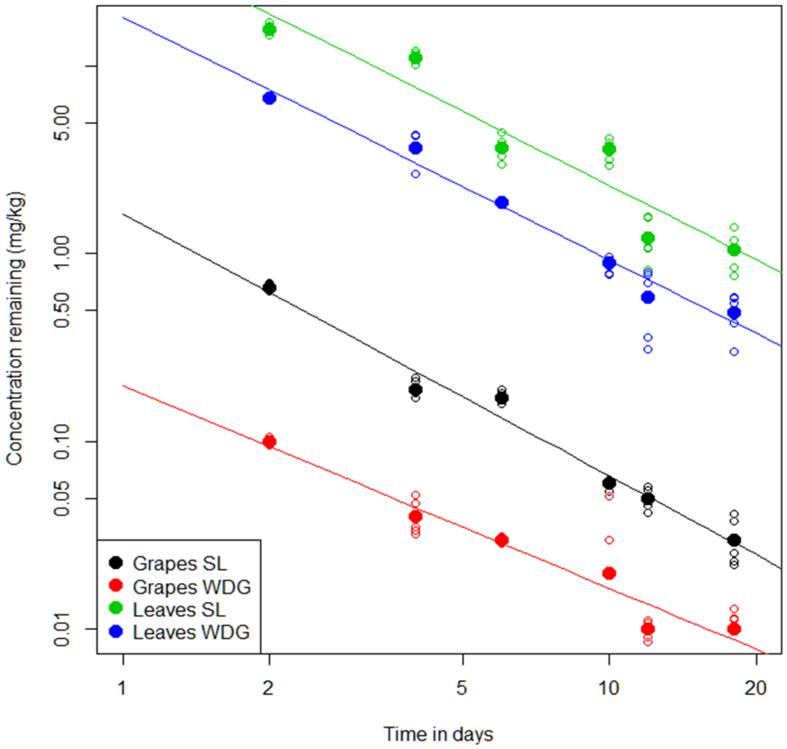

Figure S2. Model of imidacloprid degradation with separate slopes and intercepts

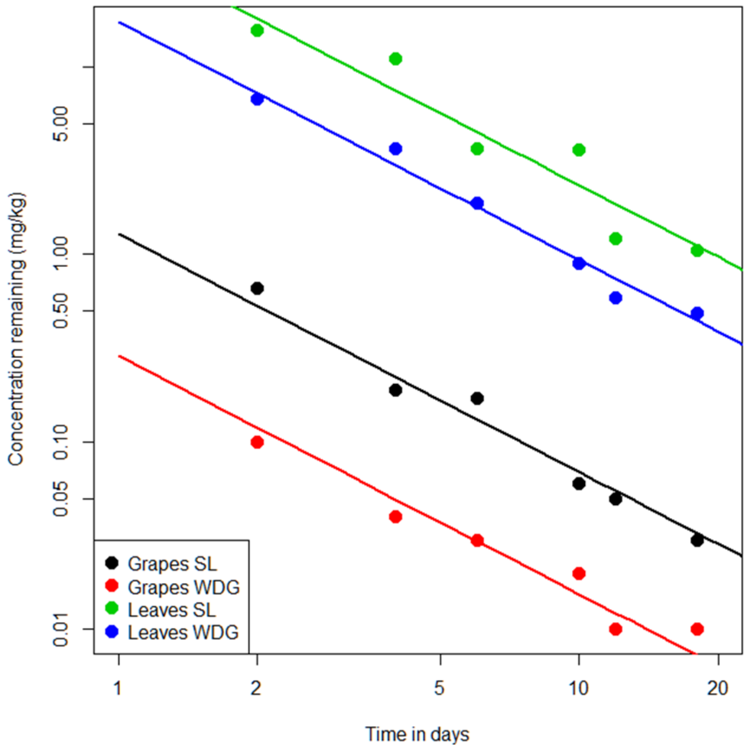

Figure 2. Model of imidacloprid degradation with pooled slope but individual intercepts

4. Discussion

The continuous change model implies that the half-life is steadily increasing with time. The rate of loss after Day 12 is very slow but was still occurring according to that model. By contrast, the two-compartment model had potentially no breakdown after Day 12.

There was no formal estimate available for the rate of breakdown beyond Day 12 for the two-compartment model. The continuous change model does give a measure for that. The continuous change model has five parameters (4 intercepts and a common slope) whereas the two compartment model requires 4 intercepts and four slopes, and four change points from one compartment to the next and four slopes within the second compartment.

Appendix A Raw data supplied

Matrix	Formulation	Time (days)	Concentration C1 (mg/kg)	Concentration C2 (mg/kg)	Concentration C3(mg/kg)	Concentration C4 (mg/kg)	Concentration C5 (mg/kg)	Mean CONCENTRATION	SD
Leaves	SL	2	14.68	15.18	16.46	14.68	17.01	15.60	0.96
Leaves	SL	4	11.99	11.75	10.2	10.08	10.97	11.00	0.78
Leaves	SL	6	3.79	3.94	2.98	3.3	4.44	3.69	0.51
Leaves	SL	10	4.1	3.16	4.14	3.87	2.93	3.64	0.50
Leaves	SL	12	1.07	0.82	1.08	1.55	1.59	1.22	0.30
Leaves	SL	18	0.76	1.39	1.07	0.85	1.18	1.05	0.23
Leaves	WDG	2	6.67	6.95	6.81	6.56	6.57	6.71	0.15
Leaves	WDG	4	3.63	2.67	3.59	4.23	4.28	3.68	0.58
Leaves	WDG	6	1.85	1.87	1.85	1.9	1.89	1.87	0.02
Leaves	WDG	10	0.97	0.97	0.77	0.96	0.79	0.89	0.09
Leaves	WDG	12	0.36	0.8	0.77	0.7	0.31	0.59	0.21
Leaves	WDG	18	0.55	0.3	0.59	0.43	0.58	0.49	0.11
Grapes	SL	2	0.69	0.65	0.63	0.63	0.7	0.66	0.03
Grapes	SL	4	0.18	0.17	0.22	0.17	0.21	0.19	0.02
Grapes	SL	6	0.16	0.16	0.19	0.18	0.17	0.17	0.012
Grapes	SL	10	0.0625	0.062	0.0546	0.0588	0.0621	0.06	0.003
Grapes	SL	12	0.0551	0.0499	0.0579	0.0416	0.0455	0.05	0.006
Grapes	SL	18	0.0232	0.0254	0.0221	0.0413	0.0379	0.03	0.008
Grapes	WDG	2	0.0976	0.0954	0.0976	0.1036	0.1059	0.10	0.004
Grapes	WDG	4	0.0321	0.0467	0.0352	0.0523	0.0338	0.04	0.008
Grapes	WDG	6	0.031	0.0291	0.0288	0.0313	0.0299	0.03	0.001
Grapes	WDG	10	0.0058	0.0064	0.051	0.0299	0.0069	0.02	0.018
Grapes	WDG	12	0.0111	0.0086	0.0109	0.0091	0.0103	0.01	0.001
Grapes	WDG	18	0.0115	0.0043	0.0128	0.0101	0.0113	0.01	0.003
